# A mediating role for mental health in associations between COVID‐19‐related self‐stigma, PTSD, quality of life, and insomnia among patients recovered from COVID‐19

**DOI:** 10.1002/brb3.2138

**Published:** 2021-04-03

**Authors:** Hosein Mahmoudi, Mohsen Saffari, Mahmoud Movahedi, Hormoz Sanaeinasab, Hojat Rashidi‐Jahan, Morteza Pourgholami, Ali Poorebrahim, Jalal Barshan, Milad Ghiami, Saman Khoshmanesh, Marc N. Potenza, Chung‐Ying Lin, Amir H. Pakpour

**Affiliations:** ^1^ Trauma Research Center and Faculty of Nursing Baqiyatallah University of Medical Sciences Tehran Iran; ^2^ Health Research Center Life Style Institute Baqiyatallah University of Medical Sciences Tehran Iran; ^3^ Health Education Department Faculty of Health Baqiyatallah University of Medical Sciences Tehran Iran; ^4^ Behavioral Sciences Research Center and Faculty of Nursing Baqiyatallah University of Medical Sciences Tehran Iran; ^5^ Health Education and Health Promotion unit Rasht Health Center,Guilan University of Medical Sciences Rasht Iran; ^6^ Rasht Health Center,Guilan University of Medical Sciences Rasht Iran; ^7^ Health, Safety and Environment Management Expert of Occupational Health of Health Center Rasht Iran; ^8^ Department of Epidemiology School of Public Health Hamadan University of Medical Sciences Hamadan Iran; ^9^ Departments of Psychiatry and Neuroscience and the Child Study Center School of Medicine Yale University New Haven CT USA; ^10^ Institute of Allied Health Sciences Department of Occupational Therapy Department of Public Health National Cheng Kung University Hospital College of Medicine National Cheng Kung University Tainan Taiwan; ^11^ Social Determinants of Health Research Center Research Institute for Prevention of Non‐Communicable Diseases, Qazvin University of Medical Sciences Qazvin Iran; ^12^ Department of Nursing School of Health and Welfare Jönköping University Jönköping Sweden

**Keywords:** coronavirus, COVID‐19, quality of life, sleep disorders, stigmatization, stress

## Abstract

**Introduction:**

Patients with COVID‐19 often suffer from psychological problems such as post‐traumatic stress disorder (PTSD) and self‐stigmatization that may negatively impact their quality of life and sleep. This study examined mental health as a potential mediating factor linking self‐stigmatization and PTSD to quality of life and sleep.

**Methods:**

Using a cross‐sectional design, 844 people who had recovered from COVID‐19 were called and interviewed. Data were collected using structured scales. Structural equation modeling was applied to assess fitness of a mediation model including self‐stigma and PTSD as independent factors and quality of life and insomnia as dependent variables.

**Results:**

Mental health, COVID‐19‐related self‐stigma, and mental quality of life were associated. Insomnia, PTSD, and COVID‐19‐related self‐stigma displayed significant direct associations (*r* = .334 to 0.454; *p* < .01). A mediation model indicated satisfactory goodness of fit (CFI = 0.968, TLI = 0.950, SRMR = 0.071, RMSEA = 0.068). Mental health as a mediator had negative relationships with COVID‐19‐related self‐stigma, PTSD, and insomnia and positive associations with quality of life.

**Conclusion:**

Mental health may mediate effects of COVID‐19‐related self‐stigma and PTSD on quality of life and insomnia. Designing programs to improve mental health among patients with COVID‐19 may include efforts to reduce negative effects of PTSD and COVID‐19‐related self‐stigma on quality of life and insomnia.

## Graphical abstract

COVID‐19 may result in having post‐traumatic stress disorder (PTSD) and self‐stigma. COVID‐19 may induce mental health problems and insomnia. Mental health mediated the association between self‐stigma and insomnia. Mental health mediated the association between PTSD and insomnia.

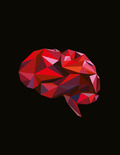

## 1 INTRODUCTION

COVID‐19 is a disease related to a novel coronavirus (SARS‐CoV‐2) that has led to a global pandemic. Given the features of rapid spread and the potential for severe respiratory symptoms, COVID‐19 may significantly impact physical health (Mohapatra et al., 2020; Wiwanitkit, 2020). This global public health issue also impacts health systems, economies, and social interactions. Many jurisdictions have implemented preventive measures such as lockdowns, spatial distancing, hand washing, and wearing of face masks (Kamenidou et al., 2020). Major efforts have emphasized limiting transmission and decreasing deaths through such public health interventions (Lahiri et al., 2020). At the time of this writing (August 12, 2020), more than 20 million people had been infected by SARS‐CoV‐2 globally (Worldometer, 2020). In Iran, a country that shortly after China began experiencing COVID‐19, between 2000 and 2500 people have been infected daily with a mortality rate of 1% to 3% (Sharifi et al., 2020).

Given the novelty of COVID‐19, precise information regarding the SARS‐CoV‐2 virus (e.g., virus transmission) is scarce, and the possibility of reinfection is not well known and under investigation (Lim, 2020). Uncertainties regarding SARS‐CoV‐2 and COVID‐19 may generate fear and insecurity, especially in vulnerable populations (Fofana et al., 2020; Reznik et al., 2020). Other factors (e.g., growing numbers of affected people, quarantines, and lockdowns) may impact psychological health, and such effects may be greater in vulnerable groups including people with preexisting conditions, older adults, children, pregnant women, and healthcare workers (Amerio et al., 2020). A recent review with a focus of COVID‐19‐related post‐traumatic stress disorder (PTSD) in healthcare workers reported that self‐blame and stigma, apart from previous mental health status, emerged as possible risk factors for PTSD (Carmassi, Foghi, et al., 2020).

During the pandemic, people (especially those with COVID‐19) may experience stress, anxiety, depression, irritability, frustration, and hopelessness (Guo et al., 2020; Serafini et al., 2020). Such mental health concerns may lead to both shorter‐ and longer‐term problems, particularly when experienced in combination with other factors such as poverty and insufficient healthcare services (Rangel et al., 2020). Data from disasters suggest emotional distress as a prevalent phenomenon in directly affected and vulnerable populations (North & Pfefferbaum, 2013).

Self‐stigma is an important factor related to mental distress during the COVID‐19 pandemic. Affected individuals may perceive themselves as being different from normal, unaffected people in terms of physical or mental abilities (Lucksted & Drapalski, 2015). People may thus classify themselves as being undesirable because of having had COVID‐19 (Bruns et al., 2020). With elevated likelihoods of self‐stigma, having had COVID‐19 may lead to social isolation, temporary unemployment, and unknown shorter‐ and longer‐term effects on health (Bruns et al., 2020; Turner‐Musa et al., 2020). Behaviors of family members, relatives, and close friends to protect individuals with COVID‐19 may exacerbate disease‐related self‐stigma (Turner‐Musa et al., 2020). People who travel from infected regions to relatively clear zones may also encounter xenophobia that may intensify self‐stigma (Cheng, 2020). Degrees of stigmatization may vary based on context and culture (Bruns et al., 2020).

Mental health concerns such as PTSD and sleep disorders are reported complications of COVID‐19 (Liu, Stevens, et al., 2020). For example, one‐fifth of 1,300 Italian participants had significant symptoms of PTSD (Castelli et al., 2020). Individuals with lower health‐related quality of life (HRQoL) were more likely to experience PTSD (Castelli et al., 2020; Liu, Stevens, et al., 2020). PTSD symptoms may relate to concerns about COVID‐19, such as infection with a potentially deadly disease and possible transmission of the disease to others (e.g., parents and children; Fekih‐Romdhane et al., 2020).

Traumatic events may negatively impact sleep quality. A recent review on sleep disorders among people locked down due to COVID‐19 in Europe indicated that individuals went to bed later and spent more time on smart phones and digital devices with poorer quality of sleep (Altena et al., 2020). Associations between stress, anxiety, and depression with sleep problems have been reported (Stanton et al., 2020). Such changes in mental health and related domains may link to reduced HRQoL, especially psychological HRQoL (Liu, Stevens, et al., 2020).

Relationships between stigmatization and PTSD with lower HRQoL and sleep problems have been identified in patients with different conditions (McCarthy et al., 2019; Wang et al., 2020). However, it is unclear how improving mental health may be effective in reducing potential negative effects of self‐stigma and PTSD on HRQoL and sleep. A mediation model can provide insight into how independent variables (self‐stigma and PTSD) may operate through a mediating factor (mental health) to influence dependent variables (HRQoL and insomnia).

Mediating roles of mental health in relationships between psychological factors and sleep problems/HRQoL have been reported (Galletta et al., 2019; Holtge et al., 2019; Pavlova et al., 2017). However, to the best of our knowledge, such investigations have never been studied among patients with COVID‐19. Therefore, the aim of the current study was to investigate whether poor mental health may mediate concerns related to infection with COVID‐19 (i.e., self‐stigma and PTSD) and outcomes such as poor sleep and HRQoL in people having recently recovered from COVID‐19.

## 2 MATERIALS AND METHODS

### 2.1 Participants and procedure

A cross‐sectional study was designed to collect data from four hospitals located in Tehran, Qazvin, and Rasht cities. A convenient sampling method was used. People having been admitted to these hospitals from February to June 2020 were eligible. Patients were contacted following hospitalization for telephone interviews to gather data. Sample size was computed to find a significant association between PTSD/self‐stigma and HRQoL/insomnia with the following parameters: *r* = .1, *α* at two‐tailed = 0.05, and a type‐2 error rate at 0.2 (i.e., power = 0.8). Inclusion criteria were: ability to speak Persian, diagnosis of COVID‐19 based on polymerase chain reaction (PCR) test and chest CT scan, at least 1 month of time having passed since discharge and absence of acute signs/symptoms of disease such as coughing, dyspnea, and fever. People with chronic disease, organ dysfunction or prior history of longstanding sleep problems or severe mental disorders such as major depression or Alzheimer's disease were excluded.

### 2.2 Measures

#### 2.2.1 Mental Health Inventory‐5

The Mental Health Inventory‐5 (MHI‐5), a short version of the MHI and MHI‐18, assessed mental health. The psychometric properties of the MHI‐5 were adequate (Cronbach's *α* = 0.775 in the present study), and the MHI‐18 has been translated into Persian with satisfactory psychometric properties (Meybodi et al., 2011). The MHI‐5 contains five items each scored along a 6‐point Likert scale with item scores ranging from 1 (all of the time) to 6 (none of the time). Higher MHI‐5 scores indicate better mental health.

#### 2.2.2 Short Form‐8

The Short Form‐8 (SF‐8) assessed physical and mental quality of life (QoL). The SF‐8 is a short version of the SF‐36 and SF‐12. The psychometric properties of the SF‐8 were adequate (Cronbach's *α* = 0.894 in the present study), and the SF‐12 and SF‐36 have been translated into Persian with satisfactory psychometric properties (Pakpour et al., 2011). The SF‐8 contains eight items with one each reflecting the eight subscales in the SF‐36 (physical functioning, role limitations due to physical health problems, bodily pain, general health perceptions, vitality, social functioning, role limitations due to mental health problems, and mental health). The items are converted into a 0–100 scale using the *T*‐score concept (i.e., mean at 50 with an *SD* of 10). Two summary scores were computed to represent physical QoL (i.e., physical composite score; PCS) and mental QoL (i.e., mental composite score; MCS). A higher score in the PCS or MCS indicates better physical or mental QoL (Yiengprugsawan et al., 2014).

#### 2.2.3 Insomnia Severity Index

The Insomnia Severity Index (ISI) assessed insomnia. The psychometric properties of the Persian ISI were adequate (Cronbach's *α* = 0.825 in the present study). The ISI contains seven items each scored along a 5‐point Likert scale with item scores ranging from 0 to 4. Higher ISI scores indicate more severe insomnia (Lin et al., 2020).

#### 2.2.4 Short‐Form PTSD Checklist‐5

The Short‐Form PTSD Checklist‐5 (SF‐PCL‐5) assessed the PTSD (Zuromski et al., 2019). The psychometric properties of the Persian SF‐PCL‐5 were adequate (Cronbach's *α* = 0.812 in the present study). The SF‐PCL‐5 contains four items each scored along a 5‐point Likert scale with item scores ranging from 0 to 4. Higher PCL‐5 scores indicate more severe PTSD. The SF‐PCL‐5 scale was developed to predict PTSD diagnoses using the PCL‐5 ≥28 threshold. The Persian version of PCL‐5 has previously translated in Persian with acceptable psychometrics (Sadeghi et al., 2016).

#### 2.2.5 Self‐Stigma Scale‐Short modified for COVID‐19

The Self‐Stigma Scale‐Short (SSS‐S) was modified to assess COVID‐19‐related self‐stigma. Although the SSS‐S was designed to understand self‐stigma in people with mental health concerns and immigrant and sexual‐orientation minority groups (e.g., gay, lesbian, and bisexual individuals), it has been used more widely given its structure. Specifically, the term describing the stigmatized group (e.g., with mental illness) may be replaced, and the present study used, “with COVID‐19 infection” to describe the potentially stigmatized group. The psychometric properties of the SSS‐S were adequate (Cronbach's *α* = 0.936 in the present study). The SSS‐S contains five items each scored along a 4‐point Likert scale with item scores ranging from 1 to 4. Higher SSS‐S scores indicate greater self‐stigma (Chang et al., 2018).

### 2.3 Statistical analysis

Descriptive statistics were employed to understand participants' characteristics. Means with SDs were applied to continuous variables and frequencies with percentages to categorical variables. Means and SDs were also used to identify central tendencies. Pearson correlations were calculated to assess associations.

Structural equation modeling (SEM) using full information maximum likelihood estimators was applied to examine the mediation model. The SEM was performed with bias‐corrected confidence intervals using 5,000 bootstrapping iterations. Before assessing direct and indirect effects in the mediation model, several fit indices were applied to determine whether the mediation model was supported. Apart from a nonsignificant *χ* ^2^ test, comparative fit index (CFI) >0.9, Tucker–Lewis index (TLI) >0.9, standardized root mean square residual (SRMR) <0.08, and root mean square error of approximation (RMSEA) with a 90% confidence interval (CI) were used to examine the model, with these thresholds indicative of a satisfactory fit (Fung et al., 2019). Direct effects of PTSD and COVID‐19‐related self‐stigma on outcomes (physical QoL, mental QoL and insomnia) were tested using *t* tests. Indirect effects of mental health in the association of independent variables (PTSD and COVID‐19‐related self‐stigma) and outcome variables (physical QoL, mental QoL and insomnia) were tested using a bootstrapping method. A mediation effect was considered supported if the 95% confidence intervals of the coefficients calculated from the bootstrapping samples did not cover 0. Analyses were conducted using IBM SPSS 24.0 (IBM Corp) or AMOS 24.0 (IBM Corp).

### 2.4 Ethical statement

The study protocol was approved by the ethics committee of Baqiyatallah University of Medical Sciences (IR.BMSU.REC.1399.187), and informed consent was obtained from participants at enrollment.

## 3 RESULTS

Among 844 participants with a mean age of 45.91 (*SD *= 13.40) years, approximately two‐thirds (*n* = 579; 68.6%) were male. Over three‐fourths (*n* = 660; 78.2%) were currently married and most had comorbidities (*n* = 704; 83.4%). The average time from discharge was 37.2 (*SD *= 29.4) days (Table 1).

**TABLE 1:** Characteristics of the study participants (*N* = 844)

	Mean (*SD*) or *n* (%)
Age (year)	45.91 (13.40)
Gender (male)	579 (68.6)
Marital status	
Single	92 (10.9)
Married	660 (78.2)
Divorced/widowed	92 (10.9)
Educational status	
Illiterate	74 (8.8)
Primary school	90 (10.7)
Secondary	282 (33.4)
University	398 (47.2)
Current Smoking status (yes)	82 (9.7)
Employment status	
Employed	444 (52.6)
Unemployed	88 (10.4)
Retired	126 (14.9)
Housekeeper	132 (15.6)
Student	54 (6.4)
Comorbidity (yes)	704 (83.4)
Time from discharge (days)	37.2 (29.4)

All study variables were correlated with one another with moderate or greater magnitudes (Table 2). Correlations were in anticipated directions: MHI‐5, PCS, and MCS were positively correlated with each other (*r* = .388 to 0.769; *p* < .01); ISI, PCL‐5, and COVID‐19‐related SSS‐S were positively correlated with each other (*r* = .334 to 0.454; *p* < .01); and MHI‐5, PCS, and MCS were negatively correlated with ISI, PCL‐5, and COVID‐19‐related SSS‐S (*r* = −.688 to −0.376; *p* < .01).

**TABLE 2:** Descriptive statistics and Pearson correlation matrix of the key study variables

	Mean	*SD*	*r*					
			MHI−5	PCS	MCS	ISI	SF‐PCL−5	SSS‐S
MHI−5	61.36	19.40	–	.388	.585	−.480	−.547	−.417
PCS	92.94	23.09		–	.769	−.439	−.532	−.376
MCS	59.66	22.90			–	−.534	−.688	−.466
ISI	10.35	6.60				–	.454	.334
SF‐PCL−5	5.13	3.51					–	.446
SSS‐S	1.78	0.71						–

Note

All *p*‐values < .01.

Abbreviations: ISI, Insomnia Severity Index; MHI‐5, Mental Health Inventory‐5; SF‐8 MC, Short Form‐8 mental component score; SF‐8 PCS, Short Form‐8 physical component score; SF‐PCL‐5, Short‐form PTSD Checklist‐5; SSS‐S, Self‐Stigma Scale‐Short.

The mediation model showed a good fit as reflected by satisfactory fit indices (CFI = 0.968, TLI = 0.950, SRMR = 0.071, RMSEA = 0.068), except for the significant *χ* ^2^ test (*p* < .001). PTSD was directly associated with physical QoL (unstandardized coefficient [*B*] = −24.61; *SE* = 2.26; *p* = .002), mental QoL (*B* = −17.87; *SE* = 1.92; *p* = .003), and insomnia (*B* = 0.51; *SE* = 0.09; *p* = .003) and indirectly associated with physical QoL (*B* = −11.20; bootstrapping *SE* = 6.53; 95% CI = −26.19, −1.16), mental QoL (*B* = −1.44; bootstrapping *SE* = 0.69; 95% CI = −2.81, −0.12), and insomnia (*B* = 0.24; bootstrapping *SE* = 0.05; 95% CI = 0.14, 0.35) via mental health. COVID‐19‐related self‐stigma was directly associated with physical QoL (*B* = −3.17; *SE* = 1.00; *p* = .002), mental QoL (*B* = −2.33; *SE* = 0.68; *p* = .001), and insomnia (*B* = 0.12; *SE* = 0.05; *p* = .011) and indirectly associated with physical QoL (*B* = −2.50; bootstrapping *SE* = 1.31; 95% CI = −6.41, −0.75), mental QoL (*B* = −0.55; bootstrapping *SE* = 0.30; 95% CI = −1.27, −0.06), and insomnia (*B* = 0.09; bootstrapping *SE* = 0.02; 95% CI = 0.04, 0.14) via mental health (Table 3).

**TABLE 3:** Models that tested mediation effects relating to insomnia and sleep quality

	Unstand. Coeff.	*SE* or (Bootstrapping *SE*)	*t*‐value or (Bootstrapping LLCI)	*p*‐value or (Bootstrapping ULCI)
Mediation effects on physical QoL				
Direct effect of PTSD on physical QoL	−24.61	2.26	10.89	.002
Direct effect of self‐stigma on physical QoL	−3.17	1.00	3.17	.002
Indirect effect of PTSD on physical QoL (via mental health)	−11.20	(6.53)	(−26.19)	(−1.16)
Indirect effect of self‐stigma on physical QoL (via mental health)	−2.50	(1.31)	(−6.41)	(−.75)
Mediation effects on mental QoL				
Direct effect of PTSD on mental QoL	−17.87	1.92	9.31	.003
Direct effect of self‐stigma on mental QoL	−2.33	0.68	3.42	.001
Indirect effect of PTSD on mental QoL (via mental health)	−1.44	(0.69)	(−2.81)	(−.12)
Indirect effect of self‐stigma on mental QoL (via mental health)	−0.55	(0.30)	(−1.27)	(−.06)
Mediation effects on insomnia				
Direct effect of PTSD on insomnia	0.51	0.09	5.67	.003
Direct effect of self‐stigma on insomnia	0.12	0.05	2.40	.011
Indirect effect of PTSD on insomnia (via mental health)	0.24	(0.05)	(0.14)	(.35)
Indirect effect of self‐stigma on insomnia (via mental health)	0.09	(0.02)	(0.04)	(.14)

Note

Age, gender, education, smoking status, time from discharge and marital status were adjusted for the model.

Abbreviations: LLCI, lower limit in 95% confidence interval; PTSD, posttraumatic stress disorder; QoL, quality of life; ULCI, upper limit in 95% confidence interval; Unstand. Coeff, unstandardized coefficient.

## 4 DISCUSSION

This study examined the hypothesis that mental health would mediate relationships between COVID‐19‐related self‐stigma and PTSD and HRQoL and insomnia in patients who recovered from COVID‐19. Findings supported mediation effects. The direct and significant relationships between the independent variables and outcome measures were confirmed, and SEM results showed that a model in which mental health is considered as mediator between study variables had good fit indices. Moreover, both components of HRQoL (i.e., physical and mental components) as well as insomnia were associated with the mediator. Therefore, addressing mental health as an entity through which associations between COVID‐19‐related self‐stigma/PTSD link to HRQoL/insomnia may help design effective intervention strategies to prevent negative psychological effects of COVID‐19 on HRQoL and sleep.

Prior studies have examined the extent to which mental health may have a mediating role. For example, Galletta et al.( 2019) examined the mediation effect of mental health between sense of coherence and physical HRQoL in 209 people with chronic diseases. They found that despite a nonsignificant direct association between sense of coherence and physical HRQoL, the indirect effect of the independent variable on HRQoL through mental health was significant. They concluded that a sense of coherence as a marker of HRQoL may be considered as a psychological process influencing mental health, which in turn may affect physical HRQoL as well. Choi et al.( 2016) identified a mediating role of mental health linking symptom severity and HRQoL in 519 patients with urinary tract infections. In this study, all three subscales of the Depression, Anxiety and Stress Scale‐21 displayed similar mediation effects in relationships between symptom severity and HRQoL. Thus, they suggested that when improving HRQoL among such patients, the role of mental health in designating effective programs should be considered along with physical relief of symptoms. Yuan et al.( 2020) used a similar design to ours in examining and identifying a partially mediating role of mental health between sleep quality and HRQoL in older adults in China. While these studies used different scales to assess mental health, the findings resonate with the current ones in suggesting a mediating role of mental health.

A point that differentiates our study from those mentioned above is that we used a brief and complete scale (i.e., MHI‐5) to assess mental health. We believe that although the MCS of the HRQoL may be considered as a measure to assess mental health, it is a part of the measure specifically designed to measure HRQoL. We thus believe that our use of an independent scale to assess mental health may be better to assess independent relationships.

As hypothesized, mental health, and HRQoL measures were negatively associated with insomnia and PTSD. Therefore, patients with COVID‐19 who had sleep problems and significant PTSD symptomatology experienced poor HRQoL and mental health. Poor sleep quality has been observed in people with COVID‐19. Among hospitalized patients in intensive care wards of the Wuhan Union Hospital, COVID‐19 patients with poor sleep quality compared to those with better sleep quality had lower recovery rates, poorer immune system functioning and longer hospital stays, negatively impacting their HRQoL (Zhang et al., 2020). Among patients who had recovered from COVID‐19 and were followed for 8 weeks after discharge, more than 85% had sleep problems including changes in subjective sleep quality, sleep latency, sleep duration, and daytime function. More than half of participants were also identified with significant generalized anxiety (Yang et al., 2020). As good sleep may promote better immune system function in COVID‐19 patients, early interventions to improve sleep may be helpful.

Among 675 people who were discharged following COVID‐19 treatment, PTSD and self‐stigma were significant concerns (Liu, Baumeister, et al., 2020). Patients also reported considerable perceived discrimination that generated stress and anxiety for them. Similarly, we found that the association between COVID‐19‐related self‐stigma and PTSD may lead to poorer mental health in general and poorer HRQoL generally, although these measures should be evaluated longitudinally to confirm such relationships. Therefore, finding solutions to target these relationships by decreasing self‐stigma and improving the mental health of patients by reducing stress and anxiety may help improve HRQoL and limit sleep problems among this population. This may be especially important for people with psychiatric problems. Specifically, these patients have been reported to experience more severe sequelae after a COVID‐19 infection than people in the general population. Therefore, specific attention should be paid to this vulnerable population. Indeed, recent research has found that patients with psychiatric problems had high levels of mental health burden (including PTSD and insomnia) when they were exposed to lockdowns and spatial distancing (Carmassi, Bertelloni, et al., 2020).

The current study has limitations. First, we collected data from only four hospitals that admitted patients with COVID‐19. Therefore, our findings may not be generalizable to those who received care in their homes or those with milder symptoms. Second, the study examined a convenient sample using telephone interviews. Third, the survey was cross‐sectional and causality cannot be inferred. Fourth, the survey was subject to recall and other biases. Fifth, discharged patients were contacted about 1 month after discharge and COVID‐19‐related effects on outcomes (HRQoL, insomnia) may change over time. Hence, following such patients for longer periods may help to better understand associations between study variables over time.

In conclusion, our results supported the hypothesis that mental health is a mediator between COVID‐19‐related self‐stigma/PTSD and poor HRQoL/insomnia. Therefore, considering mental health concerns in intervention or prevention measures when helping patients with COVID‐19 may provide more effective approaches to decrease complications and potential negative consequences of the disease, especially on their HRQoL and sleep. Future studies conducted within other jurisdictions may help examine the generalizability of the findings and understand mechanisms underlying associations between physical and psychological health.

## CONFLICT OF INTEREST

Dr. Potenza has consulted for and advised Opiant Pharmaceuticals, Idorsia Pharmaceuticals, AXA, Game Day Data, and the Addiction Policy Forum; has received research support from the Mohegan Sun Casino, the Connecticut Council on Problem Gambling, and the National Center for Responsible Gaming; has participated in surveys, mailings or telephone consultations related to drug addiction, impulse control disorders, or other health topics; and has consulted for law offices and gambling entities on issues related to impulse control or addictive disorders. The other authors report no financial relationships with commercial interests.

## AUTHOR CONTRIBUTIONS

HM, MS, HS, HRJ, and AHP designed the study and contributed to prepare the initial draft of the manuscript. CYL and MNP critically reviewed the manuscript. Others contributed to collect data and entering data in the analysis software. All authors studied the manuscript and provided their comments on it.

### PEER REVIEW

The peer review history for this article is available at https://publons.com/publon/10.1002/brb3.2138.

## Data Availability

Author elects to not share data.
